# Missing the forest (plot) for the trees? A critique of the systematic review in tobacco control

**DOI:** 10.1186/1471-2288-10-34

**Published:** 2010-04-25

**Authors:** Laura J Rosen, Michal Ben Noach, Elliot Rosenberg

**Affiliations:** 1 Dept. of Health Promotion, School of Public Health, Sackler Faculty of Medicine, Tel Aviv University, POB 39040, Ramat Aviv 69978, Israel; 2 Dept. of Statistics, Faculty of Exact Sciences, Tel Aviv University, POB 39040, Ramat Aviv 69978, Israel; 3 Dept. of Occupational Medicine, Israel Ministry of Health, Ben Tabai 2, Jerusalem 93591, Israel

## Abstract

**Background:**

The systematic review (SR) lies at the core of evidence-based medicine. While it may appear that the SR provides a reliable summary of existing evidence, standards of SR conduct differ. The objective of this research was to examine systematic review (SR) methods used by the Cochrane Collaboration ("*Cochrane*") and the Task Force on Community Preventive Services ("the *Guide*") for evaluation of effectiveness of tobacco control interventions.

**Methods:**

We searched for all reviews of tobacco control interventions published by Cochrane (4^th ^quarter 2008) and the *Guide*. We recorded design rigor of included studies, data synthesis method, and setting.

**Results:**

About a third of the Cochrane reviews and two thirds of the Guide reviews of interventions in the community setting included uncontrolled trials. Most (74%) Cochrane reviews in the clinical setting, but few (15%) in the community setting, provided pooled estimates from RCTs. Cochrane often presented the community results narratively. The Guide did not use inferential statistical approaches to assessment of effectiveness.

**Conclusions:**

Policy makers should be aware that SR methods differ, even among leading producers of SRs and among settings studied. The traditional SR approach of using pooled estimates from RCTs is employed frequently for clinical but infrequently for community-based interventions. The common lack of effect size estimates and formal tests of significance limit the contribution of some reviews to evidence-based decision making. Careful exploration of data by subgroup, and appropriate use of random effects models, may assist researchers in overcoming obstacles to pooling data.

## Background

At the core of evidence-based medicine, with its hierarchy of evidence and guidelines, lies the systematic review (SR). The potential of the SR to "make order out of scientific chaos" [1] has attracted researchers, policy makers, journal editors, and funders [2]. The simplicity of the core idea - to search for all relevant articles of a defined nature, followed by quantitative synthesis when possible has strong inherent appeal. Like evidence-based medicine, the more recent evidence-based public health also relies heavily on the systematic review. But attempting to accurately summarize evidence in public health has particular challenges.

One major challenge for the reviewer of public health interventions concerns inclusion criteria for original studies. Reviews in medicine often include only randomized controlled trials (RCTs). In public health, however, RCTs may be more difficult to perform [3]. Legislation and taxation, two classic public health approaches, which are implemented on a population-wide basis, are not easily amenable to assessment by randomized or even non-randomized controlled methods. Other public health interventions are community-based and are also often evaluated without the benefit of a randomized approach.

Thus, in deciding which level of evidence to include in SRs of public health intervention effectiveness, reviewers are presented with a difficult choice: restrictive inclusion criteria may exclude all or most of the existing evidence, thereby potentially missing true effects, while more liberal inclusion criteria may include studies with weaker, bias-prone study designs which may incorrectly show effectiveness.

A second challenge pertains to information synthesis. In medicine, the dose of a given medication or the details of a procedure may differ between studies, yet the interventions may be more similar than different. In public health, there is a greater possibility for heterogeneity between interventions, and the differences between their interventions may exceed the similarities. Hence the increased difficulty in deciding about the methods of synthesis of the findings of the various studies included in SRs.

Both of these challenges have been discussed previously [3]. However, no single solution has received general acceptance.

This paper compares the methods used in producing SRs of tobacco control, which were performed by the Cochrane Collaboration ("Cochrane") [4]and the Task Force on Community Preventive Services ("the Guide") [5]. The Cochrane Collaboration is "dedicated to making up-to-date, accurate information about the effects of healthcare readily available worldwide. It produces and disseminates systematic reviews of healthcare interventions and promotes the search for evidence in the form of clinical trials and other studies of interventions." [4]

The *Guide*'s mandate is to "recommend interventions to improve the performance of healthcare systems; interventions implemented in community settings such as schools, worksites, and community organizations, and interventions applied to entire communities." [[5], p. ix] In order to do this, the *Guide *develops conceptual models linking determinants of public health problems, interventions for change, intermediate outcomes such as behavioral change, and health. Understanding the "big picture" of cause and effect drives *Guide *decisions about which interventions to research; then the *Guide *performs "systematic review(s) to provide scientific evidence of the effectiveness of interventions. Recommendations are explicitly linked to this evidence and are therefore evidence-based." [[5], P. xxvi].

Consequently, both Cochrane and the *Guide *produce systematic reviews, though only the Guide consistently produces recommendations.

Cochrane is an international organization, while the work of the Task Force is based in, and geared towards, the United States. Cochrane reviews are performed by individual researchers throughout the world, subject to the rigorous demands of a Cochrane editorial review board, while *Guide *reviews are commissioned and operate under the auspices of evidence-based practice centers in the United States.

SRs from both organizations are used in the United States and internationally (US Department of Health and Human Services 2004, US Department of Health and Human Services 2004 2006, Agency for Healthcare Research Quality 2008, National Institutes of Health 2008, World Health Organization 2008, National Institute for Clinical Excellence 2008, Institute of Medicine 2008) for policy, reimbursement, and guideline setting, and are an important part of the evidence base for tobacco control policy.

The specific objectives of the present study were to (a) determine whether inclusion criteria for rigor of study design, and synthesis methods, differed between the two organizations; (b) to determine whether level of rigor differed by study setting (clinic or community), and (c) to explore how these differences may impact the utility of the reviews for health policymakers.

## Methods

We examined all reviews published by Cochrane (Cochrane 2008 4^th ^quarter, online) [4] the *Guide*, [5-7] pertaining to tobacco control. Each review was categorized by the level of design of the included studies, setting, and method of data synthesis. All information was extracted independently by two authors of this paper (LR and MBN). Disagreements were resolved by discussion.

Settings were defined as Clinical (interventions aimed at individuals in a clinical setting), Clinical system (interventions involving the healthcare system, such as subsidies for cessation medications or provider training), Community (interventions aimed at individuals in a community setting), or Mixed (trials involving interventions in two or more of the above).

To each SR, we assigned the level of evidence of the study with the least rigorous design that was included in the review. Level 1 indicated that all original studies included in the review used a randomized design. Level 2 indicated that at least one of the studies was a quasi-randomized study, and all studies were either quasi-randomized or randomized. Level 3 indicated that there was at least one non-randomized controlled trial in the set of original studies, and that all studies were either randomized, quasi-randomized, or non-randomized controlled trials. Level 4 indicated that there was at least one included original study which had an uncontrolled design (before-and-after, or interrupted time series), and that all studies were either randomized or quasi-randomized or controlled or uncontrolled.

We categorized the methods of synthesis used in the SRs as "meta-analysis," "median," or "narrative." We used the term "meta-analysis" to denote reviews in which data were synthesized using a standard statistical technique for combining outcomes. In those reviews, estimates of the intervention effect (in terms of odds ratios or relative risks) were presented with a measure of statistical significance (p-value). Those reviews which were summarized using the "median" method provided the median of the set of estimated effects from the individual studies, without an accompanying p-value. Both the meta-analysis and median methods are considered quantitative methods of synthesis in this paper. Reviews summarized with the narrative method of synthesis provided descriptive summaries of results, without an estimate of an overall intervention effect.

## Results

### Cochrane

We located the following 49 reviews, of which 46 were completed:

*C1: Acupuncture and related interventions for smoking cessation*.

C2: Antidepressants for smoking cessation

C3: Anxiolytics for smoking cessation

C4: Aversive smoking for smoking cessation

C5: Biomedical risk assessment as an aid for smoking cessation

C6: Cannabinoid type 1 receptor antagonists (rimonabant) for smoking cessation C7: Clonidine for smoking cessation

C8: Community interventions for preventing smoking in young people

C9: Community interventions for reducing smoking among adults

C10: Community pharmacy personnel interventions for smoking cessation

C11: Competitions and incentives for smoking cessation

C12: Enhancing partner support to improve smoking cessation

C13: Exercise interventions for smoking cessation

C14: Family and carer smoking control programmes for reducing children's exposure to environmental tobacco smoke

C15: Family-based programmes for preventing smoking by children and adolescents

C16: Group behaviour therapy programmes for smoking cessation

C17: Healthcare financing systems for increasing the use of tobacco dependence treatment

C18: Hypnotherapy for smoking cessation

C19: Impact of tobacco advertising and promotion on increasing adolescent smoking behaviours

C20: Individual behavioural counselling for smoking cessation

C21: Interventions for preoperative smoking cessation

C22: Interventions for preventing tobacco sales to minors

C23: Interventions for preventing tobacco smoking in public places

C24: Interventions for promoting smoking cessation during pregnancy

C25: Interventions for smokeless tobacco use cessation

C26: Interventions for smoking cessation in hospitalised patients

C27: Interventions for tobacco cessation in the dental setting

C28: Interventions for waterpipe smoking cessation

C29: Interventions to reduce harm from continued tobacco use

C30: Lobeline for smoking cessation

C31: Mass media interventions for preventing smoking in young people

C32: Mass media interventions for smoking cessation in adults

C33: Mecamylamine (a nicotine antagonist) for smoking cessation C34: Nicobrevin for smoking cessation

C35: Nicotine receptor partial agonists for smoking cessation

C36: Nicotine replacement therapy for smoking cessation

C37: Nursing interventions for smoking cessation

C38: Opioid antagonists for smoking cessation

C39: Physician advice for smoking cessation

C40: Psychosocial interventions for smoking cessation in patients with coronary heart disease

C41: Quit and Win contests for smoking cessation

C42: Relapse prevention interventions for smoking cessation

C43: School-based programmes for preventing smoking

C44: Self-help interventions for smoking cessation

C45: Silver acetate for smoking cessation

C46: Telephone counselling for smoking cessation

C47: Tobacco cessation interventions for young people

C48: Training health professionals in smoking cessation

C49: Workplace interventions for smoking cessation

There were 44 completed reviews in the Cochrane library's Tobacco Addiction Group^c1-c23, c25-c27, c29, c31-c32, c34-c40, c42-c49 ^(one of these^c23 ^was temporarily withdrawn; an earlier version was therefore used in the present analysis), three planned reviews which were not performed because no original studies were found for inclusion (these reviews were excluded from further analyses),^c28, c30, c34 ^one smoking-related review in the Pregnancy Group, ^c24 ^and one smoking-related review in the Heart Group.^c41 ^The studies had been carried out in Clinical settings (19/46, 41.3%),^c1-c7, c18, c21, c26, c27, c33, c35-c40, c45 ^Clinical systems (2/46, 4.3%),^c17, c48 ^Community (13/46, 28.3%),^c8-c12, c19, c22, c23, c31, c32, c41, c43, c49 ^and Mixed settings (eg, settings for different included studies were different, or a specific intervention took place both in the clinical and community setting) (12/46, 26.1%).^c13-c16, c20, c24, c25, c29, c42, c44, c46, c47 ^Most reviews included designs at Level 1 (26/46, 56.5%).^c1-c7, c10, c12, c13, c15, c16, c18, c21, c25, c33, c35-c38, c40, c42-c45, c48 ^A fair number included designs at Levels 2 or 3 (14/46, 30.4%),^c8, c9, c11, c14, c20, c24, c26, c27, c29, c31, c39, c41, c46, c47 ^while a minority of reviews included designs at Level 4 (6/46, 13.0%). ^c17, c19, c22, c23, c32, c49 ^Results were synthesized using meta-analysis in most (30/46, 65.2%) of reviews,^c1-c7, c11, c12, c16-c18, c20, c24-c27, c29, c35-c40, c42-c47 ^with the remainder (16/46, 34.8%) summarized using a narrative approach.^c8-c10, c13-c15, c19, c21-c23, c31-c33, c41, c48, c49 ^

**Figure 1:**
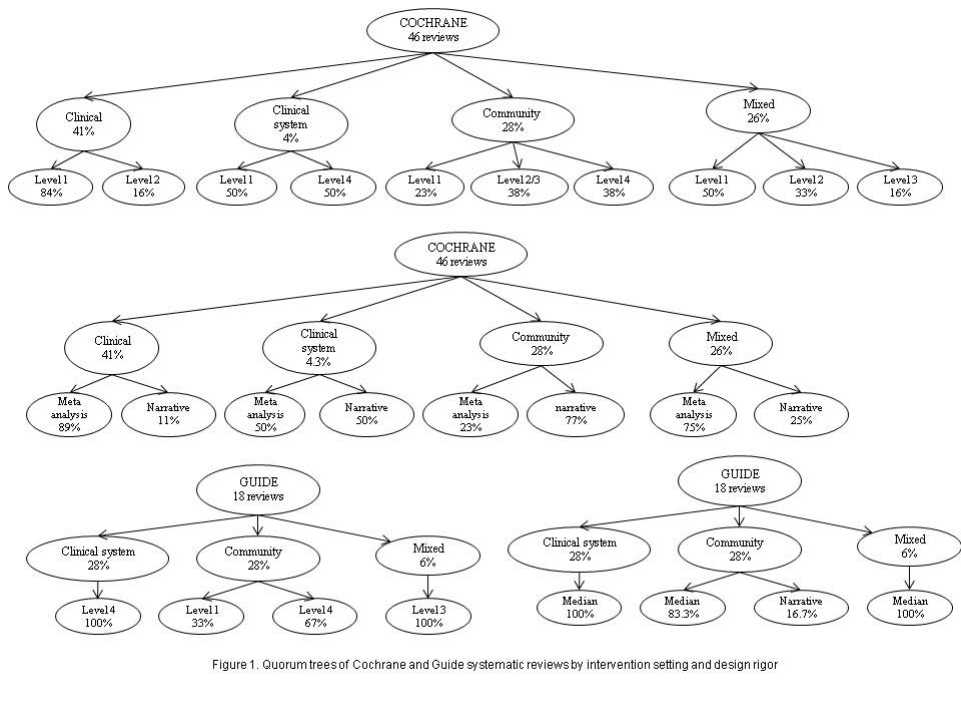
**Quorum trees of Cochrane and Guide systematic reviews by intervention setting and design rigor**.

Tables 1, 2, 3 present the distributions of the SRs by level of study design, methods of data synthesis and setting. The information is illustrated in Figure 1 as Quorum Trees [8]. Of the 19 Cochrane reviews of interventions in clinical settings, most (16/19, 84.2%) included Level 1 design types, while the remainder (3/19, 15.8%) included Level 2 design types. Meta analysis was used to synthesis the data in most cases (17/19, 89.5%). Most (14/19, 73.7%) reported statistically pooled results from RCTs, and 89.5% (17/19) reported pooled results from either RCTs or CTs.

**Table 1:** Distribution of reviews by reviewer, setting, and level of study design

Level*	Setting** N (%)				
	Clinical	Clinical System	Community	Mixed	Total
*COCHRANE*					
Level 1	16 (84)	1 (50)	3 (23)	6 (50)	26
Level 2	3 (16)	0 (0)	0 (0)	4 (33)	7
Level 3	0 (0)	0 (0)	5 (38)	2 (16)	7
Level 4	0 (0)	1 (50)	5 (38)	0 (0)	6
All levels	19 (100)	2 (100)	13 (100)	12 (100)	46
*GUIDE*					
Level 1	0 (0)	0 (0)	4 (33)	0 (0)	4
Level 2	0 (0)	0 (0)	0 (0)	0 (0)	0
Level 3	0 (0)	0 (0)	0 (0)	1 (100)	1
Level 4	0 (0)	5 (100)	8 (67)	0 (0)	13
All levels	0 (0)	5 (100)	12 (100)	1 (100)	18

* Level: Level 1 (only RCTs included in review), Level 2 (At least one quasi-randomized study included in review), Level 3 (At least one controlled trial included in review), Level 4 (At least one non-controlled trial, such as interrupted time series, included in review).

**Setting: Clinical (interventions aimed at individuals in a clinical setting), Clinical system (interventions involving the healthcare system, such as subsidies for cessation medications or provider training), Community (interventions aimed at individuals in the community), or Mixed (included trials involving interventions in two or more of the above).

**Table 2:** Distribution of reviews by reviewer, setting, and method of data synthesis

Synthesis Method*	Setting** N (%)				
	Clinical	Clinical System	Community	Mixed	All
*COCHRANE*					
Meta-analysis	17 (89)	1 (50)	3 (23)	9 (75)	30
Median	0 (0)	0 (0)	0 (0)	0 (0)	0
Narrative	2 (11)	1 (50)	10 (77)	3 (25)	16
All levels	19 (100)	2 (100)	13 (100)	12 (100)	46
*GUIDE*					
Meta-analysis	0.0 (0)	0 (0)	0 (0)	0 (0)	0
Median	0.0 (0)	5 (100)	10 (83.3)	1 (100)	16
Narrative	0.0 (0)	0 (0)	2 (16.7)	0 (0)	2
All levels	0.0 (0)	5 (100)	12 (100)	1 (100)	18

* Synthesis Method: Meta-analysis (indicating a pooled statistic), calculation of the median (median of effect sizes taken without any accompanying p-value), or narrative.

** Setting: Clinical (interventions aimed at individuals in a clinical setting), Clinical system (interventions involving the healthcare system, such as subsidies for cessation medications or provider training), Community, or Mixed (included trials involving interventions in two or more of the above).

**Table 3:** Distribution of reviews by reviewer, setting, and methodological criteria

Combined methodological criteria*	Setting** N (%)				
	Clinical	Clinical System	Community	Mixed	All
*COCHRANE*					
RCTs with pooling	14 (74)	0 (0)	2 (15)	4 (33)	20
RCTs/CTs with pooling	3 (16)	0 (0)	1 (8)	5 (42)	9
Other	2 (11)	2 (100)	10 (77)	3 (25)	17
All levels	19 (100)	2 (100)	13 (100)	12 (100)	46
*GUIDE*					
RCTs with pooling	0 (0)	0 (0)	3 (25)	0 (0)	3
RCTs/CTs with pooling	0 (0)	0 (0)	0 (0)	1 (100)	1
Other	0 (0)	5 (100)	9 (75)	0 (0)	14
All levels	0 (0)	5 (100)	12 (100)	1 (100)	18

* Combined methodological criteria: RCTs with pooling (Randomized controlled trials only, with quantitatively pooled results), RCTS/CTs with pooling (Randomized controlled trials or Controlled Trials only, with quantitatively pooled results), Other (any study design, any type of synthesis)

** Setting: Clinical (interventions aimed at individuals in a clinical setting), Clinical system (interventions involving the healthcare system, such as subsidies for cessation medications or provider training), Community, or Mixed (included trials involving interventions in two or more of the above).

Of the 13 reviews of interventions which took place in the community setting, design types were split between Level 1 (3/13, 23.1%), Level 2 or 3 (5/13, 38.5%), and Level 4 (5/13, 38.5%). Most (10/13, 76.9%) reviews of community interventions used the narrative approach for synthesis. Few (2/13, 15.4%) reported pooled results from RCTs, or from RCTs or CTs (3/13, 23.1%).

### The Guide

The Guide conducted 21 SRs which formed the basis for their recommendations [5-7]. These are listed below:

G1: Increase unit price

G2:Mass media education campaigns in combination

G3:Restricting minor's access: Community mobilization plus other

G4:Laws directed at retailers (alone)

G5:Laws directed at minor's purchase, possession, use (alone)

G6:Enforcement (alone)

G7:Retailer education with enforcement (alone)

G8:Retailer education without enforcement

G9:Community education regarding Minors' access (alone)

G10:Increase unit price

G11:Reduce costs

G12:Telephone plus multicomponent

G13:Mass media education plus other

G14:Mass media education - cessation

G15:Mass media education - contests

G16:Healthcare provider reminder system

G17:Healthcare provider reminder plus provider education

G18:Healthcare provider education alone

G19:Healthcare provider feedback

G20:Smoking bans

G21:Community education regarding environmental tobacco smoke in the home

Three reviews lacked original studies and were excluded from further analysis, ^g5, g7, g9 ^leaving a total of 18 completed reviews. Twelve Guide reviews (66.7%) concerned interventions taking place entirely in the community, ^g1-g4, g6, g8, g10, g13-g15, g20, g21 ^five (27.8%) concerned interventions within clinical systems, ^g11, g16-g19 ^and one (5.6%) took place in a mixed environment. ^g12^

Four included studies with Level 1 designs, ^g6, g8, g15, g21 ^one had Level 3 design, ^g12 ^and thirteen included Level 4 designs (72.2%).^g1-g4, g10-g11, g13-g14, g16-g20 ^The majority (16/18, 88.9%)^g1-g4, g6, g8, g10-g13, g16-g21 ^were synthesized using the median, and a few (2/18, 11.1%) ^g14, g15 ^were synthesized using the narrative technique.

Of the 12 Guide reviews which took place in the community, one third (4/12) included Level 1 designs and two thirds (8/12) included Level 4 designs. Most (10/12, 83.3%) were summarized using a median, while the remainder (2/12, 16.7%) were summarized narratively. Of the 5 Guide reviews which took place in the clinical system setting, all were Level 4 designs and were summarized using a median.

## Discussion

Our study illuminates differences in conduct of SRs on tobacco control between Cochrane and the Guide, as well as differences in levels of rigor used for study of interventions conducted in the clinical versus the community settings. These findings support recent statements regarding standards of SR conduct in medical and public health settings [9]. In a departure from other work [3] on the topic, it quantifies empirical use of different approaches to study design inclusion criteria and methods of information synthesis.

In the community setting, Cochrane reviews were less likely than *Guide *reviews to include uncontrolled trials (Cochrane: 38%, *Guide*: 67%). Findings from Cochrane reviews were quantitatively synthesized less frequently than were findings from *Guide *reviews (Cochrane: 23%, *Guide*: 83%).

Comparisons between clinical and community settings were only possible for the Cochrane reviews, as the *Guide *did not assess interventions which take place in clinical settings [5]. Nearly all (84%) of the Cochrane reviews on clinical interventions included only RCTs, while a just one quarter of its SRs of community-based interventions did so. Most Cochrane SRs of clinical interventions utilized pooled estimates of RCTs (74%), while the majority of SRs of community interventions did not (83%,).

The preference for controlled over uncontrolled trials found in community-based Cochrane reviews (62%) versus community-based Guide reviews (33%) may reflect the continued influence of the vision of Archie Cochrane, who called in 1979 for "assembly of a *critical summary*, of all... relevant *randomized controlled trials*." While recent Cochrane statements support this vision [10], other declarations are more consistent with the fact that most (77%) of Cochrane community-based SRs were not restricted to RCTs. The website of the Tobacco Addiction Group [11] includes this statement: *"Reviews will, in general, be restricted to randomized or quasi-randomized studies unless randomization is likely to be impractical for the intervention studied, in which case Controlled Before and After studies and Time Series studies may also be considered*." Declarations by the Health Promotion and Public Health (HPPH) Field [12], which became a Cochrane group in 2007, stand in stark contrast to the original vision: "*The HPPH Field does not contend that all health promotion and public health interventions must be justified by randomized controlled trials ... The criteria used to select studies *should primarily reflect the questions being answered in the review, *as opposed to any predetermined hierarchy*." While this group does not directly influence the editorial policy of the Tobacco Addiction Group, some population-based tobacco control interventions may come under its purview in the future.

Our findings identify two main limitations that may hinder the evidence-based policymaker in prioritizing interventions aimed at controlling tobacco use. The first relates to the assessment of effectiveness and the second to the assessment of effect magnitude.

Several issues affect the assessment of effectiveness of interventions. First, the synthesis of information by the narrative technique, used frequently by Cochrane, does not provide quantitative assessment of effectiveness. Cochrane's use of the narrative review is generally explicitly justified by statistical considerations: either difficulties in synthesizing data from cluster randomized trials^c10 ^or heterogeneity. ^c8, c9, c13, c14, c19, c21, c22, c23, c31, c32, c41, c49^

Difficulties in synthesis due to clustering should not pose a problem in the future, as appropriate statistical software such as RevMan™ is now available [13]. Heterogeneity [10] is a more complex issue. Heterogeneity can be "clinical" (referring to characteristics of the population or the intervention), "methodological" (referring to differences in methods used in the different studies), or "statistical" (referring to differences in the direction or magnitude of the results). It is always important to explore differences in results by using subgroup analyses. Sometimes the reasons for differences in results are due to clinical or methodological differences between the trials. In those cases, in may be best to present statistical summaries by subgroup. Heterogeneity of the results explained by methodological differences may indicate bias. When heterogeneity is present and cannot be explained by clinical or methodological factors, it may be appropriate to use a random effects, rather than fixed effects, model. While the fixed effects approach asks the question "What is the best estimate of the (one) true effect of the intervention?" the random effects model asks a different question: "What is the average effect of the intervention, in its different forms and over different target populations?" Even in situations where the first question is irrelevant (if it is assumed that different forms of an intervention have different levels of effectiveness in different populations, and so a single true effect does not exist), the second question may be of substantial interest to policy makers. RevMan and other meta analytic programs have options for running random effects as well as other models, and reviewers should consider their use, particularly when significant heterogeneity is present.

Assessment of effectiveness performed by the *Guide *is problematic for a different reason. The median synthesis technique employed has no accompanying statistical assessment (p-value). Thus, these reviews lack the common denominator of effectiveness used as the statistical basis for all of evidence-based medicine. The Guide [5] states that when synthesizing results, it prefers "simplicity of calculation." A publication by Task Force members [3] noted that this decision was part of a trade-off which allowed many reviews to be done, "despite serious deficiencies in reporting in primary studies." They predict that future editions of the Guide will include more meta-analyses.

Related challenges exist with respect to assessment of effect magnitude. Reviews summarized narratively do not provide point estimates of effectiveness. Estimates of effect magnitude from reviews which are quantitatively synthesized, which include both randomized and nonrandomized trials, and which are not explored by subgroup, may be difficult to interpret. Previous work has shown that estimates of benefit derived by reviews which included only randomized trials show less benefit than those from reviews which included less rigorous study designs [14]. The lack of assessment of effectiveness and effect magnitude lessen the quality of the information currently delivered to the policy maker.

One possible solution, to overcome the problems due to weaker or different study designs, as well as different levels of quality of implementation (such as high drop-out rates or known biases) of some community-based studies, would be to routinely stratify analyses by methodological rigor and/or quality of conduct of included studies. Comparisons of effect sizes for the same type of intervention, under different conditions of study design and conduct quality, would then be possible (assuming sufficient quantity and variety of original studies in the SRs). Exploring differences in study results as a function of differences in methodological rigor and/or implementation quality of the original studies could contribute to the current discussion of how best to perform SRs of interventions in community settings.

This report is limited in several ways. Issues relating to external validity, essential for estimating potential population impact [15], are not investigated. We could not address the effects of methodological rigor on conclusions, because the topics of the individual studies differed. (That subject is more fully explored in a separate paper, and is limited to the seven pairs of studies regarding similar topics [16].) Further, analyses are limited to SRs done by Cochrane and the *Guide*. Though we are unable to infer findings of this study to other SRs on tobacco control interventions, the issues raised regarding assessment and estimation of effectiveness are likely to be relevant in other SRs and public health topics.

## Conclusions

Policy makers should be aware that SR methods differ, even among leading producers of SRs, and among settings studied. The traditional SR approach of using pooled estimates from RCTs is employed frequently for clinical but infrequently for community-based interventions. The common lack of effect size estimates and formal tests of significance limit the contribution of some reviews to evidence-based decision making. Careful exploration of data by subgroup, and appropriate use of random effects models, may assist researchers in overcoming obstacles to pooling data.

## List of Abbreviations

SR: Systematic Review; RCT: Randomized Controlled Trial; CT: Controlled Trial; Cochrane: Cochrane Collaboration; The Guide: Task Force on Community Preventive Services;

## Competing interests

The authors declare that they have no competing interests.

## Authors' contributions

The article was conceived and drafted by LJR. ER contributed to the writing. LJR and MBN extracted the data. LJR, ER, and MBN edited the article for critical content and approved the final version of the manuscript.

## Pre-publication history

The pre-publication history for this paper can be accessed here:

http://www.biomedcentral.com/1471-2288/10/34/prepub
